# Association between social engagement decline and cognitive function changes: mediating effect of depressive symptoms

**DOI:** 10.1007/s40520-024-02897-2

**Published:** 2024-12-27

**Authors:** Ping Ni, Hongxiu Chen, Xiuying Hu

**Affiliations:** Innovation Center of Nursing Research and Nursing Key Laboratory of Sichuan Province, West China Hospital, Sichuan University/West China School of Nursing, Sichuan University, Chengdu, 610041 PR China

**Keywords:** Social engagement, Cognitive decline, Depressive symptoms, Older adults, CHARLS

## Abstract

**Background:**

Cognitive decline is a significant public health problem worldwide, but little is known about social engagement’s impact on cognitive changes over time. This study aimed to explore the relationship between social engagement decline and cognitive function change in Chinese adults, and to analyze the effect of changes in depressive symptoms on this relationship.

**Methods:**

Participants were selected from the China Health and Retirement Longitudinal Study. Multiple linear regression was conducted to determine the association between social engagement decline and cognitive function changes, and a three-step method was used to test the mediating role of changes in depressive symptoms.

**Results:**

During the four-year follow-up, participants’ overall cognitive function decreased by an average of 0.41 points (3.0%). The decline in social engagement was significantly associated with subsequent poorer global cognitive function (Model 1: β = − 0.060, *P* =.005; Model 2: β = − 0.056, *P* =.009), and changes in depressive symptoms partially mediated this effect, accounting for 15.3% of the total effect in Model 1 and 13.8% in Model 2. Model 1 adjusted for sociodemographic characteristics, and Model 2 adjusted for health-related factors based on Model 1.

**Conclusion:**

When developing programs for cognitive improvement in middle-aged and older adults, measures to enhance social engagement should be considered. This may not only reduce depressive symptoms but also help to minimize cognitive decline.

**Supplementary Information:**

The online version contains supplementary material available at 10.1007/s40520-024-02897-2.

## Introduction

According to the WHO, in 2019, there were 1 billion people over 60 years old worldwide. This number will increase to 1.4 billion by 2030 and 2.1 billion by 2050 [1]. As the number of older people increases, the prevalence of many age-related health problems, such as cognitive impairments, also increases [2]. People with cognitive impairments have a higher risk of developing dementia later in life than their peers [3]. The global number of people with dementia is expected to reach 150 million by 2050 [4]. Dementia is currently the seventh leading cause of death worldwide and one of the leading causes of disability and dependency among older adults [5]. Therefore, research into methods to slow cognitive decline and prevent dementia is currently a significant public health focus. Diet, exercise, learning, cardiovascular health, and social engagement (SE) influence cognitive function [6]. There has been much research on non-pharmacological approaches to improving cognitive function through diet, exercise, and cognitive training [7–9], but fewer studies have focused on SE and cognition.

Social engagement refers to active participation in community or society activities, such as leisure activities, volunteering, and social groups [10]. More SE is associated with better intrinsic capacity and is essential to successful aging [11, 12]. However, the SE of older adults in East Asian countries is unsatisfactory. A survey in China shows that 32.2% of people have inactive or less active SE [13]. Similarly, the participation rate of Japanese old people in “voluntary activities” and “board member or secretary activities” is only about 30% [14]. People living in nursing homes have more opportunities for SE, but a survey of nursing homes in Korea shows that the percentage of SE among older adults does not exceed 60% [15]. SE is positively correlated with executive function and episodic memory [16, 17]. Older adults with better preserved cognitive function have higher SE levels than those with mild cognitive impairment [18]. Therefore, the Lancet Commission on Dementia Prevention, Intervention, and Care recommends increasing SE in later life to help prevent or delay the onset of dementia [19]. In addition, SE affects cognitive changes. Individuals with low SE at young ages experience more significant memory loss over time [20]. However, SE also changes over time. Some studies show that SE in older adults decreases further with age [21–23]. However, most studies on SE and cognition are cross-sectional, and few focus on the effects of longitudinal SE changes on cognitive function.

Depressive symptoms commonly accompany symptoms of cognitive decline in older adults [24]. The prevalence of depression in mild cognitive impairment people is 25% in the community samples and 40% in the clinic samples [25]. Patients with depression often present with more severe cognitive impairment [26], and increased depressive symptoms lead to a higher risk of dementia [27]. Thus, intervening in depressive symptoms in patients with cognitive impairment has the potential to improve cognitive function, for example, with the help of pharmacotherapy [28], exercise [29], psychotherapy [30], and multi-component non-pharmacological interventions [31]. Notably, promoting SE may also improve depressive symptoms. Old adults with high levels of SE are less likely to experience depressive symptoms [32]. This is because self-esteem, sense of control, and perceived support availability during SE can improve physical and mental health [33]. Given the correlations between SE and cognition, depression and cognition, and SE and depression, we speculate that depressive symptoms may be one of the psychological pathways that explain the correlation between SE and cognition.

Based on the literature and the identified research gap, we proposed two hypotheses for this study: (1) decline in social engagement is associated with poorer cognitive function, and (2) changes in depressive symptoms mediate SE decline and cognitive function changes. Therefore, this study was designed to (1) describe the effects of SE decline on cognitive function changes in Chinese middle-aged and older adults, and (2) examine the role of changes in depressive symptoms in the relationship between the effects of SE decline on cognitive function change.

## Methods

### Study setting and participants

Data were collected from the 2011 (wave 1) and 2015 (wave 3) China Health and Retirement Longitudinal Study (CHARLS) (https://charls.charlsdata.com/pages/data/111/zh-cn.html). Participants in these two waves were the same cohort. CHARLS is a population-based, nationally representative longitudinal survey conducted by the National School of Development at Peking University. CHARLS is harmonized with the US Health and Retirement Study (HRS), which aims to gather a broad dataset of personal health information, social information, and economic data for geriatric and health policy research. CHARLS participants are mainly Chinese residents aged 45 and older and their spouses, with no upper age limit [34]. CHARLS was approved and organized by Peking University’s Institutional Review Board (IRB00001052-11015). All participants provided their written informed consent before they completed the survey. A total of 17,708 participants from 10,257 households were enrolled in the first survey round through multistage probability sampling. The details of the sampling methods used have been reported previously [35].

Of 17,708 participants at baseline, 15,566 participants were excluded because of (1) age < 45 years old, (2) extreme body mass index (BMI; BMI < 16 or BMI > 40), and (3) a lack of main variables (SE, depressive symptoms, and cognitive function). Ultimately, a total of 2,242 individuals were included in this study, and we analyzed baseline data from these individuals in 2011 and follow-up data from 2015.

### Assessment of cognitive function

Cognitive function was calculated using two categories: episodic memory and mental status [36]. Episodic memory was measured with a word recall test. The examiner read a list of ten random words and then asked the participants to recall as many words as possible immediately afterward (immediate recall). Ten minutes later, the participants were asked to recall the same list of words (delayed recall). Episodic memory scores were calculated as the average number of words recalled immediately and after a delay (scores ranged from 0 to 10) [36]. Mental status can be measured with tests of orientation, calculation, and visuoconstruction. To test orientation, the participants were asked the year, month, day, season, and day of the week (scored from 0 to 5). To test calculation, participants completed serial subtractions of 7 from 100 five times (scored from 0 to 5). To test visuoconstruction, participants redrew an overlapping pentagon that was shown to them (scored from 0 to 1). The mental status score was generated by summing participants’ scores on these three tests (total score from 0 to 11) [37]. Global cognition was defined as the total episodic memory and mental status score (scored from 0 to 21) [38, 39]. In this study, the change in cognitive function was expressed as the difference between cognitive function scores in 2015 and 2011.

### Assessment of social engagement

Two questions were used to measure SE. The first question was, “Have you done any of these activities in the last month?” This question was multiple choice and included twelve options, with the first eleven options being specific activities and the twelfth question being none of these. If participants chose any of options 1–11 on the first question, they were required to answer the second question, which was “Frequency of activity in the last month.” The second question included three options: (1) Almost daily; (2) Almost every week; and (3) Not regularly. SE was scored as follows: if the participant chose option 12 on the first question, SE was scored as 0. If the participant chose options 1–11 on the first question, then options (1), (2), and (3) on the second question were scored as 3, 2, or 1, respectively. Each year’s SE score was the sum of the scores for each activity. This measurement was used and validated in Yang et al.’s [40] study. In our study, SE decline was defined as a participant’s SE score in 2015 being lower than their score in 2011.

### Assessment of depressive symptoms

Depressive symptoms were measured with the ten-item Center for the Epidemiological Studies of Depression Short Form (CESD-10) scale. CESD has been widely used in Chinese. The CESD-10 has good reliability and validity among Chinese middle-aged and older adults, with a Cronbach alpha of 0.815, and the CHARLS data supports the two-factor model for the entire sample [41]. The CESD-10 consists of ten items, and participants are asked to answer the frequency of the symptoms described in each item during the past week. Each item has four options, with options (1) through (4) corresponding to scores of 0, 1, 2, or 3, respectively. The fifth question and the eighth question were scored in reverse. The total score ranges from 0 to 30, with higher scores indicating more severe depressive symptoms [42]. In this study, a change in depressive symptoms was expressed as the difference between the 2015 CESD-10 score and the 2011 CESD-10 score.

### Other covariates

Potential covariates were selected based on previous studies to control other factors influencing cognitive function [38, 43, 44]. These included sociodemographic characteristics (age, gender, marital status, education, and Hukou type) and health-related factors (BMI, chronic diseases, smoking, drinking, midday napping, activities of daily living [ADL], and instrumental activities of daily living [IADL)].

China’s Hukou system, also known as the household registration system, is a unique institutional feature of migration in China. Hukou types were divided into three categories. BMI [45] and midday napping time [46] were classified into four categories. The measure of functional ability was based on the performance of ADL and IADL. ADL consists of six items, such as eating and dressing [47]. IADL included preparing hot meals, taking medications, managing money, shopping for groceries, cleaning the house, and using the telephone [48]. Data on using the telephone were not available for Wave 1 participants. Therefore, to ensure the comparability of the data, this study used five questions to measure IADL. ADL disability was defined as difficulty with one or more ADL activities. Likewise, IADL disability was defined as difficulty with one or more IADL activities [49, 50] (Table 1).

**Table 1:** Categorization of the covariates

Covariates	Categorization
Marital status	Married, separated, divorced, widowed, never married
Educational level	Elementary school and below, middle school, high school, university and above
Hukou types	Agricultural, non-agricultural, unified resident
BMI	< 18.5 kg/m^2^, 18.5–23.9 kg/m^2^, 24–27.9 kg/m^2^, ≥ 28.0 kg/m^2^
Comorbidity	0, 1, ≥2
Smoking	Yes, no
Drinking	Yes, no
Napping time	0 min/day, 0–30 min/day, 30–90 min/day, ≥ 90 min/day
ADL disability	Yes, no
IADL disability	Yes, no

Abbreviations: BMI, body mass index; ADL, activities of daily living; IADL, instrumental activities of daily living

### Statistical analysis

Continuous variables are expressed as mean ± standard deviation (SD) or median (interquartile range, IQR). Categorical variables are expressed numerically (percentages). A paired t-test was conducted to assess the differences in participants’ global cognitive function between 2015 and 2011. Multiple linear regression models were used to analyze the relationship between SE decline and cognitive function changes. To assess potential confounders, we adjusted for the covariates. Model 1 adjusted for sociodemographic characteristics (age, gender, marital status, education, and Hukou type); Model 2 adjusted for health-related factors (BMI, number of chronic diseases, smoking, drinking, midday napping time, ADL disability, and IADL disability) based on Model 1. To verify whether changes in depressive symptoms contribute to the effects of SE decline on cognitive function changes, we used a three-step method to test the mediating effect of changes in depressive symptoms [51]. Additionally, multiple imputation was used to recover covariate information for regression to help minimize bias [52]. In the sensitivity analysis, we referred to Rist et al.’s [53] study to classify cognitive function change as a dichotomous variable; i.e., the worst 10% of the cohort’s cognitive decline distribution was defined as cognitive decline. Binary logistic regression analysis was applied at this stage.

All data analyses were performed with SPSS 26.0 (IBM, Armonk, NY, USA). A two-sided *P* of < 0.05 was considered statistically significant.

## Results

### Participant characteristics

Of the 2,242 participants included in the analysis, 1,302 (58.1%) were male; the median age was 55 years (IQR: 49, 62 years), and the median BMI was 23.8 kg/m^2^ (IQR: 21.7, 26.1 kg/m^2^). The participants’ characteristics are shown in Table 2.

**Table 2:** Baseline characteristics of the participants (*n* = 2,242)

Characteristics	*n* (%)
Age (years) 45–65 ≥ 65	1861 (83.0) 381 (17.0)
Male	1302 (58.1)
Educational level	
Primary school or below	1,008 (45.0)
Middle school	688 (30.7)
High school	428 (19.1)
College or above	118 (5.3)
Marital Status	
Married	1,961 (87.5)
Separated	123 (5.5)
Divorced	23 (1.0)
Widowed	126 (5.6)
Never married	9 (0.4)
Hukou	
Agricultural	1,492 (66.5)
Non-agricultural	731 (32.6)
Unified residence	19 (0.8)
BMI (kg/m^2^) < 18.5 18.5–23.9 24.0–27.9 ≥ 28	57 (2.5) 1132 (50.5) 781 (34.9) 272 (12.1)
Number of chronic diseases	
0	774 (34.5)
1	650 (29.0)
≥ 2	818 (36.5)
Smoking	1,043 (46.5)
Drinking	965 (43.0)
Midday napping (minutes)	
0	966 (43.1)
< 30	202 (9.0)
30–90	773 (34.5)
≥ 90	301 (13.4)
ADL disability	235 (10.5)
IADL disability	227 (10.1)

Abbreviations: IQR, interquartile range; BMI, body mass index; ADL, activities of daily living; IADL, instrumental activities of daily living

### Changes in cognitive function

**Fig. 1:**
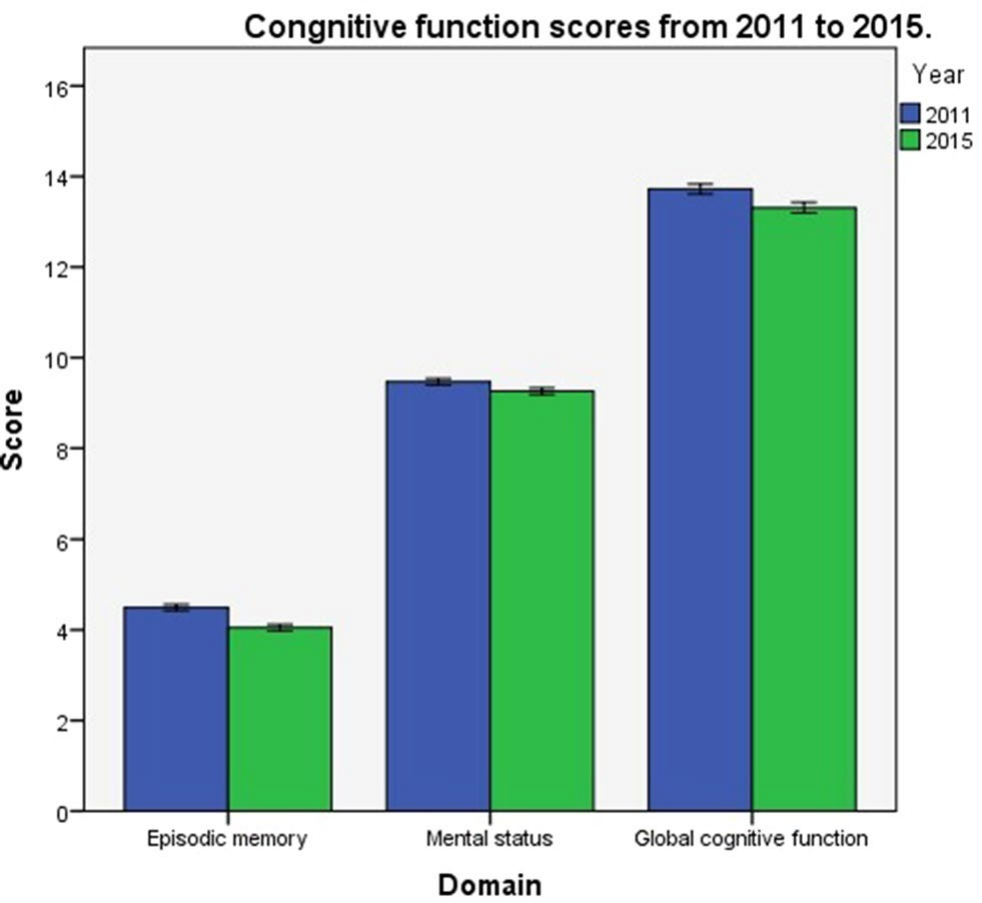
Comparison of cognitive function scores in 2011 and 2015

Participants’ scores on the cognitive function assessment changed from 2011 to 2015. The mean score for episodic memory in 2011 was 4.26 ± 1.61; that for mental status was 9.47 ± 1.72, and the mean global cognitive function score was 13.72 ± 2.66. The mean scores for these three domains in 2015 were 4.05 ± 1.67, 9.26 ± 1.80, and 13.31 ± 2.80, respectively. The global cognitive function declined by an average of 0.41 points (3.0%) during the four-year follow-up period; the difference was statistically significant (t = 7.241, *P* <.001). A comparison of cognitive function scores in 2011 and 2015 is shown in Fig. 1.

### Association between SE decline and cognitive function change

Table 3 shows the results of the multiple linear regression used to explore the relationship between SE decline and cognitive function changes. Model 1 adjusted for age, gender, education, marital status, and Hukou type. We found that SE decline was negatively associated with episodic memory changes (β =–0.042, *P* =.047), mental status changes (β =–0.047, *P* =.028), and global cognitive function changes (β =–0.060, *P* =.005). Model 2 adjusted for BMI, the number of chronic diseases, smoking, drinking, midday napping time, ADL disability, and IADL disability, based on Model 1. The results of Model 2 were similar to those of Model 1, except that episodic memory became statistically insignificant.

**Table 3:** Multiple linear regression model testing the association between SE decline and changes in cognitive function

Domain	Model 1^a^							Model 2^b^					
	B (95% CI)	SE	β	*t*	*P*	*R* ^2^		B (95% CI)	SE	β	*t*	*P*	*R* ^2^
Episodic memory change	–0.155 (–0.309, − 0.002)	0.078	–0.042	–1.986	0.047	0.013		–0.149 (–0.302, 0.005)	0.078	–0.040	–1.894	0.058	0.017
Mental status change	–0.175 (–0.331, − 0.019)	0.080	–0.047	–2.203	0.028	0.004		–0.160 (–0.316, − 0.004)	0.080	–0.043	–2.006	0.045	0.010
Global cognitive function change	–0.331 (–0.560, − 0.101)	0.117	–0.060	–2.827	0.005	0.013		–0.308 (–0.538, − 0.079)	0.117	–0.056	–2.632	0.009	0.018

Abbreviation: B, unstandardized coefficients; CI, confidence interval; β, standardized coefficients; SE, standard error

^a^ Model 1: Adjusted for age, sex, education level, Hukou, and marital status

^b^ Model 2: Adjusted for BMI, number of chronic diseases, smoking, drinking, midday napping, ADL disability, and IADL disability, based on Model 1

### Mediation effect of changes in depressive symptoms

**Fig. 2:**
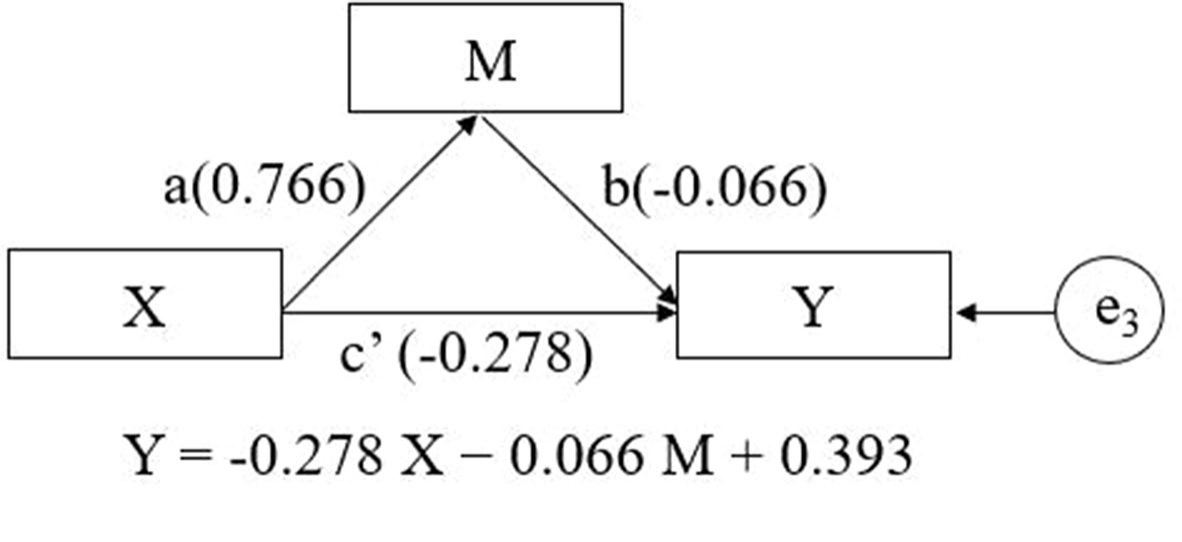
Changes in depressive symptoms examined as mediators of the association between SE decline and changes in global cognitive function in Model 1. X: SE decline. Y: Change in global cognitive function. M: Change in depressive symptoms

This study found that changes in depressive symptoms mediated the effect of SE decline on global cognitive function changes. In Path c of Model 1, SE decline affected global cognitive function changes (B = − 0.331, 95% CI [–0.560, 0.101]); in Path a, SE decline had a positive effect on changes in depressive symptoms (B = 0.766, 95% CI [0.293, 1.240]); and in Path b, changes in depressive symptoms had a negative effect on global cognitive changes (B = − 0.066, 95% CI [–0.086, − 0.047]). In Path c’, the effect of SE decline was reduced but remained significant [B = − 0.278, 95% CI (–0.506, − 0.050)]. Thus, changes in depressive symptoms were identified as a partial mediator of the effect. The mediating effect was − 0.050556 or 15.3% of the total effect (Fig. 2). Similarly, we identified changes in depressive symptoms as a partial mediator in the effect of SE decline on global cognitive function changes in Model 2, exerting a mediating effect of − 0.041958 or 13.6% of the total effect (Fig. S1).

### Sensitivity analysis

Cognitive change was analyzed as a dichotomous variable in the sensitivity analysis. The results were similar to the previous results, i.e., that SE decline was a risk factor for global cognitive function (Model 1: OR = 1.556, 95% CI [1.192, 2.031]; Model 2: OR = 1.554, 95% CI [1.182, 2.018]). More information can be found in Table S1. Changes in depressive symptoms partially mediated the effect, with a mediated effect value of 0.025278 in Model 1, accounting for 5.7% of the total effect, and 0.021312 in Model 2, accounting for 4.9% of the total effect (Fig. S2 and Fig. S3).

## Discussion

In his study, we explored the relationship between SE decline and cognitive function change in Chinese middle-aged and older adults, and we examined the effect of changes in depressive symptoms on this relationship. The results showed that Chinese middle-aged and older adults showed a decreasing cognitive function trend from 2011 to 2015. SE decline was associated with episodic memory, mental status, and global cognitive function. Changes in depressive symptoms partially mediated the effect of SE decline on global cognitive function changes. Moreover, sensitivity analysis confirmed the reliability of the results.

Our study revealed that participants’ cognitive function declined by 3% over four years. This illustrates that the prevalence of cognitive decline increases with age and is a common aspect of aging [54]. Our study also found that during the four-year follow-up period, Chinese middle-aged and older adults experienced an average decline in cognitive function of 0.41 points, which is similar to the findings of Zhang et al. [55] from CHARLS. Another study from the UK’s English Longitudinal Study of Ageing (ELSA) [56] finds that the global cognition score decreases by 0.037 for each year increase in age. The differences between the two studies may be related to the different methods used to measure cognitive function. However, both studies generally found that cognitive function further declines with age. Cognitive decline impairs older adults’ functioning and quality of life, placing burdens and stresses on families and society. However, no medications are available currently. Therefore, preventing and retarding cognitive decline is crucial.

Linear regression analyses under both models indicated that after adjusting for sociodemographic and health-related potential confounders, the association between SE decline and changes in overall cognitive function decline remained statistically significant. This suggests that SE decline is associated with overall cognitive function decline. SE has been shown to help reduce the risk of cognitive impairment and dementia [57]. However, SE changes with age, and most current studies analyze SE at a single point. Our study considered longitudinal changes in SE. We found that SE decline in middle-aged and older adults was associated with cognitive decline, as evidence from two Korean studies shows [58, 59]. Given the relationship between SE decline and cognitive change, we could develop interventions to promote SE, such as chess or dance programs [60, 61]. These interventions are beneficial as they can be accomplished by families or communities working together and are highly cost-effective. Communities’ convenience should also be considered when developing interventions to promote SE among older adults, especially those with mobility impairments [62].

This study revealed that SE decline is linked to cognitive decline, and changes in depressive symptoms are associated with both SE decline and cognitive decline. However, when changes in depressive symptoms were included in the regression model, SE decline was still associated with cognitive decline, but the strength of the association was reduced. Therefore, we identified the partial mediating role that changes in depressive symptoms play. On the one hand, SE can influence health by reducing neuroendocrine responses [63]. On the other hand, SE can provide individuals with emotional sustenance and coping assistance, alleviating the physical and emotional impacts of stressors [33]. Some have suggested that during the structured treatment of depression, the rate of response-contingent positive reinforcement must be restored to appropriate levels by changing the frequency, quality, and quantity of SE [64]. This suggests that depressive symptoms can be improved by changing individuals’ SE. Our study found this effect as well. Furthermore, depressive symptoms are strongly associated with cognitive decline in older adults. A meta-analysis of 34 studies [65] shows that depression increases the risk of cognitive decline. This study measures depression as a binary predictor; the pooled results indicate that depression is associated with an increased risk of subsequent global cognitive decline (OR = 1.36). There are three main hypotheses for this association. The first is that emotional problems may be an aetiological risk factor for cognitive decline, the second is that emotional problems may be a prodromal feature of dementia, and the last is that emotional problems and cognitive decline share common underlying neurobiological substrates [65]. Given the mediating role of depressive symptoms in the relationship of SE affecting cognitive function, it is possible that increasing SE may attenuate both depressive symptoms and cognitive decline.

This study’s strengths include using nationally representative data on Chinese middle-aged and older adults, ensuring a representative sample. Second, this study focused on longitudinal changes in SE rather than individual times. Third, the sensitivity analysis affirmed the robustness of the findings. However, the study also has some limitations. First, as in all self-reported assessments, the data on SE and depressive symptoms in this study were obtained from participants’ subjective reports and may be biased. Future studies could use a diary method to record daily SE and depression to minimize recall bias. Second, this study focused only on the level of SE and did not consider the effects of different kinds of SEs. In subsequent studies, we may explore the effects of different SEs on cognition. Finally, we did not examine the potential reverse causality of cognition on SE. Therefore, future research could explore the effect of cognitive decline on changes in SE among middle-aged and older adults.

## Conclusions

In summary, we found that the cognitive function of Chinese middle-aged and older adults tends to decline with age, and SE decline may be a risk factor for cognitive decline. Mediation analysis further suggested that changes in depressive symptoms partially mediated this effect, thereby offering a deeper understanding of how SE affects cognition. These findings suggest that when designing interventions to promote SE, the implementation approach should be adjusted according to the level of depressive symptoms in individuals to achieve better effects. Furthermore, future studies could investigate how various types of SE affect cognitive decline, offering insights for developing tailored intervention strategies.

## Electronic supplementary material

Below is the link to the electronic supplementary material.

Supplementary Material 1

## Data Availability

The National School of Development at Peking University provided the data sets in the China Health and Retirement Longitudinal Survey (http://charls.pku.edu.cn/).

## Abbreviations

CHARLS: China Health and Retirement Longitudinal Study
SE: social engagement
BMI: body mass index
CESD-10: Epidemiological Studies of Depression Short Form Scale
SDS-PAGE: Sodium dodecyl sulfate-polyacrylamide gel electrophoresis
ADL: activities of daily living
IADL: instrumental activities of daily living
SD: standard deviation
IQR: interquartile range
CI: confidence interval
