# Supplementary material for: Association between social engagement decline and cognitive function changes: mediating effect of depressive symptoms
Source: Aging Clin Exp Res. 2024 Dec 27;37(1):7. doi: 10.1007/s40520-024-02897-2 (PMC11671422; doi:10.1007/s40520-024-02897-2)
Supplement: Supplementary file 1 — Supplementary Material 1 [file 40520_2024_2897_MOESM1_ESM.docx]

**Association between social engagement decline and cognitive function changes in middle-aged and older Chinese adults: Mediating effect of depressive symptoms**

Ping Ni^a^, Hongxiu Chen^a^, Xiuying Hu^a*^

a. Innovation Center of Nursing Research and Nursing Key Laboratory of Sichuan Province, West China Hospital, Sichuan University/West China School of Nursing, Sichuan University, Chengdu, China

**^*^Correspondence:**

Xiuying Hu, Innovation Center of Nursing Research and Nursing Key Laboratory of Sichuan Province, West China Hospital, Sichuan University/West China School of Nursing, Sichuan University,

No.37 Guoxue Alley,

Chengdu, Sichuan 610041, PR China.

Phone: +86-28-85421873

Email: huxiuying@scu.edu.cn

**Supplementary Materials**

**Table S1.** Logistic regression modeling to test the relationship between social engagement decline and cognitive decline.

|  | Model 1 | | Model 2 | |
| --- | --- | --- | --- | --- |
|  | OR (95% CI) | *P* | OR (95% CI) | *P* |
| Global cognitive decline | 1.556 (1.192, 2.031) | 0.001 | 1.544 (1.182, 2.018) | 0.001 |

Abbreviations: OR, odds ratio.

**Fig. S1**


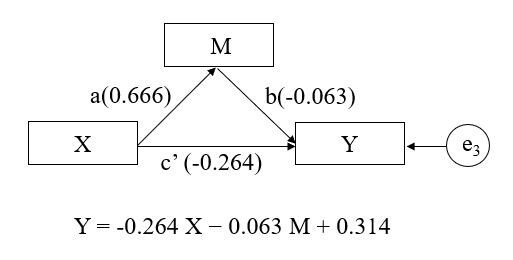


Changes in depressive symptoms examined as mediators of the association between SE decline and changes in global cognitive function in Model 2.

**Fig. S2**


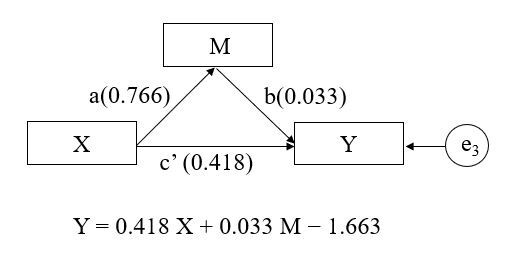


Sensitivity analysis of changes in depressive symptoms in Model 1.

**Fig. S3**

**
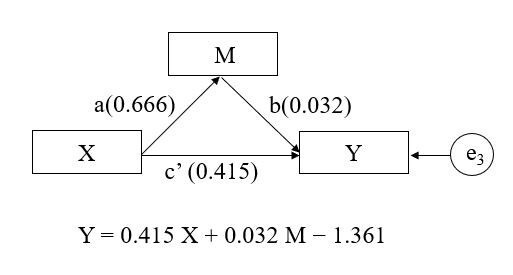
**

Sensitivity analysis of changes in depressive symptoms in Model 2.
